# Gilda: biomedical entity text normalization with machine-learned disambiguation as a service

**DOI:** 10.1093/bioadv/vbac034

**Published:** 2022-05-11

**Authors:** Benjamin M Gyori, Charles Tapley Hoyt, Albert Steppi

**Affiliations:** Laboratory of Systems Pharmacology, Harvard Medical School, Boston, MA 02115, USA

## Abstract

**Summary:**

Gilda is a software tool and web service that implements a scored string matching algorithm for names and synonyms across entries in biomedical ontologies covering genes, proteins (and their families and complexes), small molecules, biological processes and diseases. Gilda integrates machine-learned disambiguation models to choose between ambiguous strings given relevant surrounding text as context, and supports species-prioritization in case of ambiguity.

**Availability and implementation:**

The Gilda web service is available at http://grounding.indra.bio with source code, documentation and tutorials available via https://github.com/indralab/gilda.

**Supplementary information:**

Supplementary data are available at *Bioinformatics Advances* online.

## 1 Introduction

Named entity recognition (NER) and named entity normalization (NEN) are central tasks in extracting knowledge about genes, proteins, metabolites, small molecules, biological processes and other entities of interest from unstructured text in the biomedical literature. The role of NER is to identify an entity string (e.g. ‘JNK-1’) in text and of NEN, or *grounding*, is to choose an appropriate namespace and identifier corresponding to an entity string (e.g. HGNC:6881 for ‘JNK-1’).

While UniProt, ChEBI, GO and other resources make available curated lists of names and synonyms for different entity types, exact searches over their thesauri have been shown to provide insufficient coverage due to the diversity of biomedical nomenclature (Bachman *et al.*, 2018). It is further necessary to account for variability in spaces, dashes, capitalization and the expansion of certain characters (e.g. Greek letters) with respect to these lexicalizations to find relevant matches. A further challenge is ambiguity, namely, that several entities can share the same name or synonym, and it is only based on broader context (a surrounding sentence or paragraph) that one can resolve which sense is implied.

While NEN is typically integrated in systems solving more complex tasks such as full-text annotation (Wei *et al.*, 2019) and relation extraction (Valenzuela-Escárcega *et al.*, 2018), there are many ways in which NEN is useful as a standalone tool, such as in the context of interactive search interfaces (finding identifiers for search terms entered by users), data analysis (finding identifiers for data entries, e.g. drugs) and modeling (assigning identifiers for modeled concepts). Though some standalone NEN tools are available, they are limited to specific entity types or lack a principled approach to disambiguation within and across entity types (Franz *et al.*, 2021; Leaman *et al.*, 2013; Wei *et al.*, 2015).

Here, we present Gilda, a software tool and web service which integrates multiple biomedical lexical resources and implements a scored string matching algorithm [parts of which were adapted from the text tagger in Allen *et al.* (2015)] to names and synonyms across entries in these resources. Importantly, Gilda makes available over 1000 machine-learned disambiguation models for strings representing multiple ambiguous entities and can apply these models as part of the scoring process given surrounding text as context. Gilda achieves state of the art performance on several of the BioCreative VI NEN benchmarking tasks and is competitive on the rest.

Gilda is available as a public REST web-service at http://grounding.indra.bio and as an open-source Python package under a BSD license at https://github.com/indralab/gilda.

## 2 Methods

***Lexical resources*.** Gilda integrates over 1.8 million names and synonyms from 9 ontologies and lexical resources for the purpose of name matching (Table 1). In addition to standard resources such as UniProt, Gilda integrates FamPlex, a resource providing lexicalizations for protein families and complexes (shown to be a key missing component in NEN systems), as well as over two thousand lexicalizations for entities that were manually curated using a frequency-ranked list of strings mined from the literature that did not appear in other standard resources (Bachman *et al.*, 2018).

**Table 1.** Resources integrated in Gilda

Resource	Entity types	No. strings
HGNC	Genes	107 372
UniProt	Proteins	429 372
ChEBI	Small molecules, metabolites	368 222
GO	Biological processes, complexes, cellular locations	138 502
MeSH	Multiple entity types	756 359
FamPlex	Protein families and complexes	3216
EFO	Cell types, anatomy, disease, etc.	30 506
HPO	Phenotypic abnormalities	30 201
DO	Diseases	23 788

*Note*: We provide detailed references for each source in the Supplementary Material.

Each of the resources listed in Table 1 are processed to extract a list of *terms*, with each term carrying the following information, illustrated via the example of ‘MEK1’: (i) namespace (e.g. HGNC) and identifier within the namespace (e.g. 6840); (ii) text name (e.g. MEK1); (iii) type of text name (e.g. synonym); (iv) canonicalized text name (e.g. mek1); (v) standard name (e.g. MAP2K1); (vi) source (e.g. hgnc). (vii) Source namespace and identifier [optional], set if the term was created by mapping the original namespace and identifier (viii) Organism [optional], when representing a gene or protein, what organism it is specific to.

In its default configuration, Gilda does not distinguish a given gene from its protein product, and the sets of synonyms for each gene and its corresponding protein are merged. Nevertheless, Gilda can be configured such that these synonyms are kept separate under separate (e.g. HGNC and UniProt) identifiers if such customization is desired for a use case.

To allow extensions and customizations, the Gilda Python package supports instantiation with custom grounding resources, and is able to load terms from ontologies in OBO and other standard formats. A tutorial notebook demonstrating multiple examples of customization is available at https://github.com/indralab/gilda/blob/master/notebooks/custom_grounders.ipynb.

***Efficient approximate scored string matching.*** Gilda implements a grounding algorithm inspired by Allen *et al.* (2015) that allows for efficient approximate matches to any of the terms appearing in the integrated resource table. For a given entity string, the grounding process first generates relevant variants (e.g. by spelling out Greek letters) and then identifies possible matches for each variant. This involves canonicalizing the variant string then searching the resource table for the same canonicalized text name. This lookup is efficient since the service loads the resource table in memory, indexed by canonicalized text names in a hash map. A string comparison algorithm then compares the original (i.e. not yet canonicalized) entity string with each matched entry to assign a score. The string comparison algorithm takes the following into account when scoring each match: (i) dashes, hyphens and spaces; (ii) capitalization; (iii) whether the entity is possibly a plural form; (iv) the status of the term that was matched (standard name, synonym, withdrawn entry, etc.). The final score is between 0 and 1, with 1 corresponding to an exact match of a standard name. Further details of the matching and scoring procedure are described in Supplement Section S5.2.

As an example, consider the input ‘PKC-Delta’. For this input, Gilda first generates the following canonicalized variants: ‘pkc delta’, ‘pkcd’, ‘pkcdelta’, ‘pkc*δ*’. These variants are then looked up (exactly) in the grounding resource for terms with matching canonicalized text names. With these canonicalized lookups, it matches three terms overall, with the following text names: ‘PKCD’ (a match for ‘pkcd’), ‘PKCdelta’ (a match for ‘pkcdelta’) and ‘PKC*δ*’ (a match for ‘pkc*δ*’), all terms representing the human gene HGNC:9399 whose standard symbol is PRKCD. Once these terms are matched, it scores each match based on string similarity and status. In this case, both ‘PKCdelta’ and ‘PKC*δ*’ have the same string similarity score, and both have a ‘curated’ status. In contrast, ‘PKCD’ receives a higher string similarity score due to the fact that the capitalization of the suffix ‘D’ in ‘PKCD’ matches that of the input suffix ‘Delta’. However, ‘PKCD’ has a ‘synonym’ status and therefore receives an overall lower score. Ultimately, since all these matches are for the same entity (i.e. HGNC:9399), only the first highest scoring match is surfaced, in this case to the term whose text name is ‘PKCdelta’ with an overall score of 0.9936.

***Machine-learned models for context-aware disambiguation.*** Many entries integrated in Gilda’s resource table share a text name (e.g. ‘DAP4’ is a gene synonym for both DLGAP4 (DLG associated protein 4) and THAP12 (Death associated protein 4). These entries are textually equivalent and their status (i.e. synonym) is identical, therefore the scores assigned to them for a given input entity string will be the same. As of Gilda v0.9.0, there are 3662 synonyms that are shared between at least two distinct human genes and 569 synonyms that are shared between 3 or more distinct human genes.

In order to resolve these ambiguities and choose between the different senses of an entity, we used labeled articles from the biomedical literature and trained logistic regression models of the context surrounding each member of a set of ambiguous terms that share a synonym.

We formulated disambiguation as a classification problem with one classification model per ambiguous entity text. We make the one sense per discourse assumption (Gale *et al.*, 1992), that an entity text is unlikely to be used with multiple senses within the same document. With this approach to disambiguation, the key challenge is, for each ambiguous entity text, to produce a corpus of documents where the entity text appears and where each document is labeled with the sense in which the entity text is being used in that document. Gilda leverages existing annotations of documents from the Entrez Gene database (Maglott *et al.*, 2011) and MeSH (Rogers, 1963) which, for each term (gene in the case of Entrez and multiple entity types for MeSH), provide a list of associated PubMed publication records. Given the PubMed identifiers for a given entity, we obtained either full text content from PubMed Central or an abstract from PubMed, depending on availability.

**Fig. 1.**
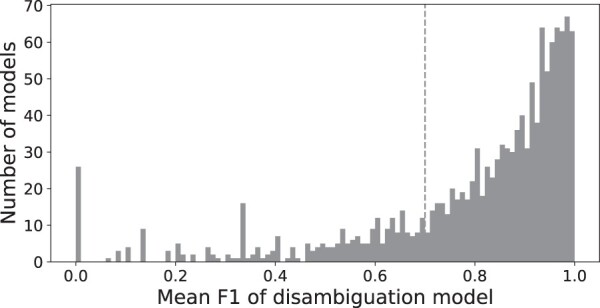
A histogram of *F*_1_ cross-validation scores for all of the trained disambiguation models. A dashed vertical line indicates the cutoff of 0.7 that was chosen for model inclusion

Initially, we identified 1287 ambiguous agent texts with senses for which a sufficient number of labeled documents were available between Entrez and MeSH. We then trained logistic regression models for each of these agent texts using term frequency-inverse document frequency (tf-idf) (Rajaraman and Ullman, 2001) vectorized unigrams and bigrams as features, performing 5-fold cross-validation in order to estimate generalization error. We evaluated model performance using the macro-averaged *F*_1_ score. For each model, we considered the mean of the macro-averaged *F*_1_ scores from each cross-validation split. A histogram of these *F*_1_ scores over all models can be seen in Figure 1. Reasons for poor performance in some cases include insufficient amounts of training data, and highly similar senses that are difficult to distinguish. We chose a cutoff of 0.7 for macro-averaged *F*_1_ scores and excluded all models with score below it from deployment.

Overall, Gilda v0.9.0 provides 1008 deployed disambiguation models. Gilda additionally integrates 172 models provided by the Adeft package (Steppi *et al.*, 2020), allowing for disambiguation of a total of 1180 ambiguous entity texts. Adeft models are classifiers that can choose between senses of the most commonly occurring acronyms in biology literature with multiple senses (e.g. ‘ER’) given surrounding text context, using a model training strategy centered around finding defining patterns (i.e. a spelled out definition of an acronym) in text. When Gilda is called with an ambiguous string to which one of the models is applicable, it uses context (for instance, a sentence or a paragraph) supplied by the user to make a prediction and adjust the scores of returned matches.

A tutorial for training such disambiguation models, in case customizations are made to Gilda, is available at https://github.com/indralab/gilda/blob/master/models/model_training.ipynb.

***Cross-species protein disambiguation.*** In addition to text as context, Gilda also takes an optional species prioritization list as input. The list can be provided directly by the user to express prior knowledge or preference for normalizing to a specific species such as human or yeast. Alternatively, in case the text to be normalized comes from a PubMed-indexed publication, MeSH annotations associated with the publication can be used to automatically derive a species preference order.

## 3 Results

***BioCreative VI benchmark.*** We benchmarked Gilda on a grounding task with the BioCreative VI Track 1 (Bio-ID) dataset (https://biocreative.bioinformatics.udel.edu/media/store/files/2017/BioIDtraining_2.tar.gz) from Arighi *et al.* (2017) (https://biocreative.bioinformatics.udel.edu/tasks/biocreative-vi/track-1/). This corpus consists of 102 717 entity texts chosen from 570 publications with a curated grounding provided for each entity text. Some of the provided groundings, however, do not refer to a specific identifier in an ontology. For example, ‘protein: GST’ appears as an entry, but it does not represent a specific grounding in an ontology. We classify such groundings as unknown and filter them out to retain only rows that refer to entries in specific namespaces such as NCBI Gene, UniProt, GO, Uberon, etc., resulting in a total of 86 574 rows. The namespaces used in the Bio-ID corpus do not align exactly with ones Gilda uses by default. For instance, Bio-ID provides gene and protein groundings to NCBI Gene and UniProt, whereas Gilda uses HGNC for human genes/proteins and UniProt for non-human genes/proteins. Similarly, Bio-ID provides groundings for tissues in Uberon whereas Gilda uses MeSH to ground tissues. We therefore applied equivalence mappings between these namespaces to allow matches to equivalent entries in different ontologies from the one used for a given row in Bio-ID. For example, one row of Bio-ID contains ‘brain’ as an entity text and provides UBERON:0000955 as its grounding; using equivalence mappings between Uberon and MeSH, we also allow MESH:D001921 as an acceptable grounding.

Table 2 shows the results of running Gilda on this benchmark. We calculated precision and recall under two conditions: a strict one in which we only accept a grounding if it is the ‘top’ match returned by Gilda, and a more permissive one in which we score a grounding as correct if ‘any’ of the matches returned by Gilda corresponds to the expected grounding, irrespective of its ranking.

**Table 2.** Precision, recall and *F*_1_ values for Gilda performance on the Bio-ID corpus by entity type

Entity type	Prec. (top)	Prec. (any)	Rec. (top)	Rec. (any)	*F*_1_ (top)	*F*_1_ (any)	Bio-ID
Cell types/cell lines	0.695	0.722	0.520	0.540	0.595	0.618	0.65
Cellular component	0.587	0.615	0.442	0.463	0.504	0.528	0.49
Human gene	0.710	0.794	0.678	0.759	0.694	0.776	0.40
Non-human gene	0.660	0.706	0.586	0.626	0.621	0.664	0.40
Small molecule	0.693	0.769	0.579	0.642	0.631	0.700	0.52
Taxon	0.677	0.707	0.517	0.540	0.586	0.612	0.76
Tissue/organ	0.690	0.690	0.436	0.436	0.534	0.534	0.51
miRNA	0.358	0.358	0.282	0.282	0.315	0.315	—
Overall	0.677	0.729	0.569	0.613	0.618	0.666	—

*Note*: Values are given both for the case where Gilda’s result is considered correct only if the ‘top’ grounding matches and the case where Gilda’s result is considered correct if ‘any’ of its returned groundings match. The right-most column (‘Bio-ID’) provides the top scoring normalization F-score reported in Arighi *et al.* (2017) (best score achieved across multiple teams/systems and multiple runs).

We compared Gilda with both the best results reported in Bio-ID (Table 2, Bio-ID column), and other published systems that reported results on one or more entity types in the Bio-ID corpus. Gilda achieved state of the art *F*_1_ for proteins [0.694 for human and 0.621 for non-human versus 0.445 from Zhou *et al.* (2020)], cellular components [0.504 versus 0.491 from Sheng *et al.* (2017)] and small molecules [0.631 versus 0.591 from Kaewphan *et al.* (2018)]. It underperformed for taxon entities [0.586 versus an average 0.623 (0.756 best) over several configurations from Dai and Singh (2018)], cells/cell lines (0.595 versus 0.740 from Kaewphan) and tissues (0.534 versus 0.633 from Kaewphan) likely due to gaps in the lexical resources in Gilda covering those entity types. Gilda’s relatively higher performance on genes (and proteins) and small molecules (including metabolites, drugs, etc.) is notable in that these entities constitute the main building blocks of biochemical pathways, while other entity types such as cell types or tissues describe biological context. Gilda—in its default configuration—is therefore particularly well suited for normalizing entities involved in biochemical mechanisms.

We found that model-based disambiguation played a role in the evaluation, affecting the resolution of 693 entries. The overall $F_{1}(\mathrm{top})$ score decreased from 0.618 to 0.614 when disabling the use of these models. Examples of strings that were correctly grounded when using disambiguation models, but were incorrectly normalized without them, include ‘ER’ (192 cases) and ‘DCs’ (43 cases).

While most previous works generated static entity-type-specific models, Gilda uses a single general model for all entity types which can be readily updated on new releases of its underlying lexical resources and extended to new tasks by incorporating additional lexical resources. However, we acknowledge that its generalization has a negative impact on Gilda’s precision in comparison to entity-type-specific models.

We noted a relatively large gap for human genes between the ‘top’ and ‘any’ conditions and found that there were two factors affecting these scores. First, we found that the Bio-ID corpus often assigns a specific protein grounding to entity texts that are ambiguous, and could refer to a protein family containing the specific protein. For example, ‘VEGF’ in Bio-ID is annotated as the specific gene VEGFA (HGNC:12680) whereas Gilda grounds it to the VEGF protein family in the FamPlex namespace, an entry which represents all human VEGFs. We found that when we accepted matches by Gilda to a FamPlex family or complex of which the Bio-ID-annotated specific protein is a member, *F*_1_-score for human genes was 0.762, significantly higher than the 0.694 reported in Table 2 where only a strict match is allowed. Second, we found that the Bio-ID corpus differentiates human and non-human forms of the same gene within the same paper. In several cases, Gilda found the expected species-specific gene but lower in the priority order (which in Gilda’s case is constructed at the paper level).

***FamPlex benchmark.*** We evaluated Gilda on a manually curated set of 300 entity texts randomly selected from relation extractions by the Reach system (Valenzuela-Escárcega *et al.*, 2018) on a corpus of 270 000 papers defined by Bachman *et al.* (2018). The corpus consists of abstracts from PubMed and (where available), full text content from PubMed Central or via the Elsevier text and data mining API covering articles specifically relevant for signaling pathways [for more details, see Table 1 of Bachman *et al.* (2018)]. We used the grounding results described by Bachman *et al.* (2018) as reference (‘Reference’ row of Table 3) to calculate precision, recall and *F*_1_ scores, and compared it with groundings produced by Gilda for the same entity strings (‘Gilda’ row of Table 3). Corresponding to the grounding logic applied in the reference data, in this setting, we ran Gilda without species-disambiguation. We found that using Gilda resulted in a relative improvement compared to the reference both in terms of precision and recall, by 4.8% and 5.3%, respectively. Similar to Bio-ID, we found that disabling the disambiguation models decreased overall performance for this benchmark: *F*_1_ dropped to 0.907 when running the benchmark without disambiguation.

**Table 3.** FamPlex benchmarking results

Trial	Precision	Recall	*F*_1_
Reference (Bachman *et al.*, 2018)	0.900	0.850	0.874
Gilda	0.944	0.895	0.919
Absolute improvement	0.044	0.045	0.045
Percentage improvement	4.8%	5.3%	5.1%

***Gilda responsiveness.*** We benchmarked the responsiveness (i.e. speed) of Gilda in three settings: (i) when run as a Python package; (ii) when run as a local web service; and (iii) when using the publicly available web service instance. We used all entity texts from the BioCreative VI Bio-ID corpus (the same corpus used for benchmarking in the previous subsection) and measured the average number of groundings performed per unit time with and without context added (i.e. additional surrounding text provided along with the entity text for disambiguation purposes). For benchmarking Gilda in the first two scenarios (when running as a local Python package or a local web service), we used a desktop PC with a 2.5 GHz Intel i9-11900 processor and 64 GB of RAM running Ubuntu 20.04 and Python 3.8.10. Results are shown in Table 4.

**Table 4.** Summary statistics on the Gilda responsiveness benchmark on the Bio-ID corpus under three scenarios (local Python package, local web app and public web app), and with and without context-based disambiguation

Scenario	Context	Requests per second
Python package	False	18794.3±7952.6
	True	18141.9±8148.1
Local web app	False	798.0±48.9
	True	584.8±78.2
Public web app	False	26.6±1.3
	True	14.8±1.6

*Note*: The mean requests served per second and its standard deviation are shown.

While usage as a local Python package was by far the fastest (over 18 thousand entity strings grounded per second), this usage mode may not be ideal in settings where startup time, memory usage and Python dependencies are undesirable. In such cases using the web application is recommended. The local web application performed better than the remote application likely due to the lack of overhead from network communication.

***Availability and integrations.*** Gilda is available as a Python package through PyPI, a Docker image through DockerHub and as a web application with a RESTful API at http://grounding.indra.bio. Gilda is integrated into INDRA (Gyori *et al.*, 2017) to ground entity texts from knowledge sources where grounding is missing and to disambiguate entity strings from text mining. Gilda is also integrated into multiple web services and a human-machine dialogue system as described below.

Gilda is used in the INDRA Database web application (https://db.indra.bio) which allows searching for statements assembled by the INDRA system (Gyori *et al.*, 2017). Here, Gilda grounding allows users to enter entity texts in non-standard form (e.g. using informal synonyms) and select an appropriate grounding used for the search, as demonstrated in Supplementary Figure S2. Gilda is also integrated into the EMMAA dashboard (https://emmaa.indra.bio) where, similar to its integration with the INDRA Database, it supports queries based on user-entered entity texts.

Gilda is used to support human-machine dialogue in the context of the CLARE system which plugs into Slack workspaces as an application. In this context, Gilda grounds named entities appearing in questions from users, a key component of interpreting natural language input and constructing responses. Supplementary Figure S3 shows an example dialogue in which Gilda is invoked to ground a human gene, a biological process and a small molecule based on non-standard synonyms.

## 4 Discussion

We presented Gilda, a NEN software and web service. Gilda’s default configuration allows it to normalize human and non-human genes/proteins, their families and complexes, small molecules, biological processes, diseases and other terms. Gilda does not attempt to distinguish between a gene and its protein product, though grounding resources for gene and protein synonyms could be separated such that this is possible. Still, scientists’ use of synonyms for genes and proteins interchangeably typically does not allow for making this distinction reliably.

Gilda can be instantiated with resources other than its built-in defaults. Challenges in this area include the fact that ontologies containing lexicalizations for named entities use different curation standards (often requiring custom processing for ingestion), and that ontologies often contain equivalent terms without providing cross-references between these terms, resulting in unresolved redundancy. It is worth noting that Gilda can play a role in improving the latter problem, by automatically detecting lexical overlaps between different ontologies and thereby proposing cross-references that can be confirmed by experts. Systematically implementing this workflow is part of future work.

Gilda’s approach of training one disambiguation model per ambiguous term requires creating numerous distinct models. However, the training process is automated, and each model is lightweight enough (both in storage size and execution time) to allow deployment as part of the Gilda software. If non-trivial ambiguities exist between entities in custom resources (i.e. ones loaded outside Gilda’s built-in ones), new disambiguation models may need to be built to allow choosing between these based on context. To facilitate this, Gilda provides sample code and tutorials for training new models. One limitation of Gilda’s disambiguation models is that they use word frequency features as attributes requiring context text to be provided. Future work will generalize features to include, for instance, known interactions between entities as context.

## Funding

This work was funded under the Defense Advanced Research Projects Agency Communicating with Computers Program [W911NF-15-1-0544] and Young Faculty Award [W911NF-20-1-0255].

*Conflict of Interest*: none declared.

## Data availability

The data underlying this article are available in the article and in its online supplementary material.

## Supplementary Material

vbac034_Supplementary_Data
