# Supplementary material for: Gilda: biomedical entity text normalization with machine-learned disambiguation as a service
Source: Bioinform Adv. 2022 May 11;2(1):vbac034. doi: 10.1093/bioadv/vbac034 (PMC9710686; doi:10.1093/bioadv/vbac034)
Supplement: vbac034_Supplementary_Data [file vbac034_supplementary_data.pdf]

5 Supplementary Material

5.1 Data sources

Gilda integrates multiple lexical resources to collect names and synonyms, as well as former names and synonyms for entities. The resources integrated in Gilda by default are as follows:

- HGNC (HUGO Gene Nomenclature Committee) (Tweedie *et al.*, 2021)
- ChEBI (Chemical Entities of Biological Interest) (Hastings *et al.*, 2016)
- UniProt (Bateman *et al.*, 2021)
- GO (Gene Ontology) (Gene Ontology Consortium, 2021)
- MeSH (Medical Subject Headings) (Rogers, 1963)
- FamPlex (Bachman *et al.*, 2018)
- EFO (Experimental Factor Ontology) (Malone *et al.*, 2010)
- HPO (Human Phenotype Ontology) (Köhler *et al.*, 2014)
- DO (Disease Ontology) (Schriml *et al.*, 2012)

5.2 String Matching and Scoring

Keys into Gilda's grounding terms are stored in a canonical form. Given a string Y, the canonical form $\mathcal{C}(Y)$ is obtained by replacing all contiguous runs of white-space characters with a single ASCII space, converting all text characters to lower case and removing all ASCII and Unicode dash characters.

Given a string X containing the raw text of an entity that is to be grounded, Gilda generates a set of lookup strings $\mathcal{L}(X)$ and searches for them among the canonicalized keys in the lexicon. One lookup string in $\mathcal{L}(X)$ is the canonical form $\mathcal{C}(X)$, but an additional set of lookup strings are included to allow for more flexible matching. Given X, lookup strings are generated for the canonical forms of each of the following strings.

- X.
- X after all ASCII and Unicode dashes have been replaced with spaces (example: "EGF-receptor" -> "EGF receptor").
- X after all spelled out Greek letters have been replaced with their single character Unicode equivalents.(example: "PKC-alpha" -> "PKCA")
- X after all spelled out Greek letters from a subset with close Latin equivalents have been replaced with the single character Latin equivalent. (example: "IKK-β" -> "IKKB")
- X after all Unicode Greek characters have been replaced with the spelled out Latin form. (example: "GSK3-β -> "GSK3-beta")
- The depluralized form of X, if X is determined to be in a possible plural form through rule-based pattern matching. (example: "RAFs" -> "RAF")

Matches are then scored based on the "status" of the matching entry in the lexicon and based on a string comparison score computed between the original uncanonicalized agent text X and the original uncanonicalized entry Y in the lexicon corresponding to the match.

The possible statuses for entries in the lexicon are listed below. Each status is assigned a numerical score encoding its priority.

- **Assertion:** A name or synonym for an entity which has been manually curated as unambiguous. *Score 4*
- **Name:** The standard name for an entity within the given ontology or lexical resource. *Score 3*
- **Synonym:** A known synonym for an entity that is listed within the given ontology or lexical resource. *Score 2*
- **Previous:** A term which was previously the standard name for an entity in a given ontology or lexical resource. *Score 1*

Given a raw agent text X and the associated raw agent text Y for a matching entry, the string comparison score $\mathcal{S}_{\text{string}}(X, Y)$, is a numerical score between 0 and 1. If the entry corresponding to Y has status score $\mathcal{S}_{\text{status}}$, and disambiguation score then the overall score for the match is given by

$$\mathcal{S}_{\text{overall}} = \frac{2\mathcal{S}_{\text{status}} + \mathcal{S}_{\text{string}}}{9}\mathcal{S}_{\text{disamb}} \quad (1)$$

which yields a numerical score between 0 and 1. Here $\mathcal{S}_{\text{disamb}}$ is the probability assigned by a disambiguation model if one was available and applied, or 1 (i.e., no change to the overall score) if disambiguation was not applied. Scoring of individual matches, i.e., the calculation of $\mathcal{S}_{\text{string}}$, follows the text tagging algorithm in Allen *et al.* (2015) and is described in detail at `http://trips.ihmc.us/TextTagger/docs/README.xhtml#sec-5.7` (see formula for "case_dash_score"). To summarize the key details here: the score is calculated as a normalized linear combination of six sub-score terms. Five of the sub-score terms penalize different patterns of capitalization mismatches between the two strings, while the sixth term penalizes dash-mismatches between the strings. The motivation to use capitalization mismatches in the score is to be able to differentiate canonical capitalization patterns that are meaningfully different from each other (e.g., the capitalization mismatch between "eGFR" and "EGFR", is different compared to the capitalization mismatch between "Egfr" and "EGFR'). For example, the largest component of the sub-score terms penalizes capitalization mismatches where a short (max. 3 characters) all caps word such as "FOR" is matched against an all lowercase word such as "for" with the goal of penalizing mismatches between short all-caps abbreviations and short regular words. Dash mismatches are also penalized but with lower weight overall since they are relatively common. More details can be found at `http://trips.ihmc.us/TextTagger/docs/README.xhtml#sec-5.7`.

5.3 Applications

This supplementary section contains figures to accompany Section 3.

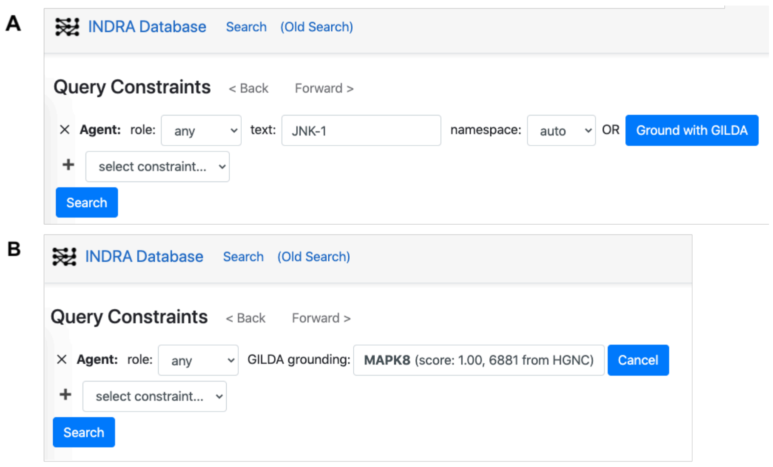

Fig. 2. Gilda is used to ground entity texts before submitting search queries to the INDRA Database at https://db.indra.bio. (A) The user enters the entity text "JNK-1", then presses the "Ground with GILDA" button. (B) Gilda returns HGNC:6881 (standard name MAPK8) as the grounding, which is then used to perform the search.

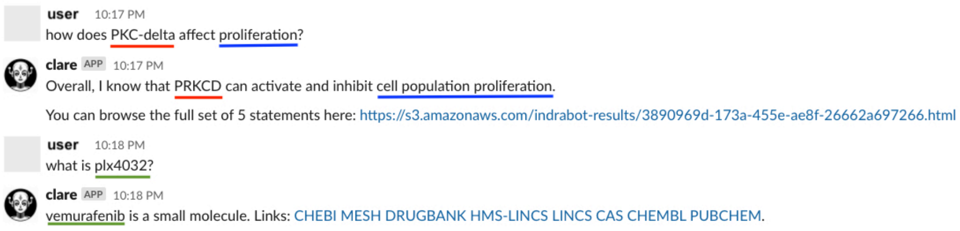

Fig. 3. Gilda is used to ground three entity texts in the context of human-machine dialogue. "PKC-delta" is grounded to HGNC:9399 with standard name PRKCD (red underline), "proliferation" is grounded to GO:0008283 with standard name cell population proliferation (blue underline), and "plx4032" is grounded to CHEBI:63637 with standard name vemurafenib (green underline).

Supplemental References

Allen, J. *et al.* (2015). Complex event extraction using drum. *ACL-IJCNLP*.

Bachman, J. A. *et al.* (2018). FamPlex: a resource for entity recognition and relationship resolution of human protein families and complexes in biomedical text mining. *BMC Bioinformatics*, **19**(1), 1–14.

Bateman, A. *et al.* (2021). UniProt: the universal protein knowledgebase in 2021. *Nucleic Acids Research*, **49**(D1), D480–D489.

Gene Ontology Consortium (2021). The Gene Ontology resource: enriching a GOld mine. *Nucleic Acids Research*, **49**(D1), D325–D334.

Hastings, J. *et al.* (2016). ChEBI in 2016: Improved services and an expanding collection of metabolites. *Nucleic Acids Research*, **44**(D1), D1214–D1219.

Köhler, S. *et al.* (2014). The human phenotype ontology project: linking molecular biology and disease through phenotype data. *Nucleic Acids Research*, **42**(D1), D966–D974.

Malone, J. *et al.* (2010). Modeling sample variables with an experimental factor ontology. *Bioinformatics*, **26**(8), 1112–1118.

Rogers, F. B. (1963). Medical subject headings. *Bulletin of the Medical Library Association*, **51**, 114–116.

Schriml, L. M. *et al.* (2012). Disease ontology: a backbone for disease semantic integration. *Nucleic Acids Research*, **40**(D1), D940–D946.

Tweedie, S. *et al.* (2021). Genenames.org: the HGNC and VGNC resources in 2021. *Nucleic Acids Research*, **49**(D1), D939–D946.
